# New case of trichorinophalangeal syndrome-like phenotype with a de novo t(2;8)(p16.1;q23.3) translocation which does not disrupt the TRPS1 gene

**DOI:** 10.1186/1471-2350-15-52

**Published:** 2014-05-02

**Authors:** Milena Crippa, Ilaria Bestetti, Mario Perotti, Chiara Castronovo, Silvia Tabano, Chiara Picinelli, Guido Grassi, Lidia Larizza, Angela Ida Pincelli, Palma Finelli

**Affiliations:** 1 Medical Cytogenetics and Molecular Genetics Lab, Istituto Auxologico Italiano, Milan, via Ariosto 13, Italy; 2 Department of Medical Biotechnology and Translational Medicine, Università degli Studi di Milano, via Viotti 3/5, Milan, Italy; 3 Medical Clinic, Hospital San Gerardo, Università di Milano-Bicocca, Monza, via Pergolesi 33, Italy; 4 Department of Pathophysiology Medical-Surgical and Transplant, Università degli Studi di Milano, Milan, via Sforza 35, Italy; 5 IRCCS MultiMedica, Sesto San Giovanni, Milan, Via Milanese 300, Italy; 6 Medical Genetics, Department of Health Sciences, Università degli Studi di Milano, Milan, via Rudini 8, Italy

**Keywords:** Reciprocal translocation, Conserved enhancer element, TRPS, *TRPS1*

## Abstract

**Background:**

Trichorhinophalangeal syndrome (TRPS) is a rare autosomal dominant genetic disorder characterised by distinctive craniofacial and skeletal abnormalities. TRPS is generally associated with mutations in the *TRPS1* gene at 8q23.3 or microdeletions of the 8q23.3-q24.11 region. However, three deletions affecting the same chromosome region and a familial translocation t(8;13) co-segregating with TRPS, which do not encompass or disrupt the *TRPS1* gene, have been reported. A deregulated expression of *TRPS1* has been hypothesised as cause of the TRPS phenotype of these patients.

**Case presentation:**

We report the clinical and molecular characterisation of a 57-year-old Caucasian woman carrying the t(2;8)(p16.1;q23.3) *de novo* balanced translocation. The proband presented with peculiar clinical features (severe craniofacial dysmorphism, alopecia universalis, severe scoliosis, mitral valve prolapse, mild mental impairment and normal growth parameters) that partially overlap with TRPS I. Mutational and array CGH analyses ruled out any genetic defect affecting *TRPS1* or genomic alteration at the translocation breakpoint or elsewhere in the genome. Breakpoint mapping excluded disruption of *TRPS1*, and revealed that the chromosome 8q23.3 breakpoint was located within the IVS10 of the long intergenic non-coding RNA *LINC00536*, at approximately 300 kb from the *TRPS1* 5’ end. Conversely, the 2p16.1 breakpoint mapped within a LINE sequence, in a region that lacks transcriptional regulatory elements. As a result of the translocation, nucleotide base pair additions and deletions were detected at both breakpoint junction fragments, and an evolutionarily conserved VISTA enhancer element from 2p16.1 was relocated at approximately 325 kb from the *TRPS1* promoter.

**Conclusions:**

We suggest that the disruption of the genomic architecture of *cis* regulatory elements downstream the *TRPS1* 5′ region, combined with the translocation of a novel enhancer element nearby *TRPS1*, might be the pathogenetic mechanism underpinning the proband’s phenotype. The clinical and genetic characterisation of the present subject allowed us to make a genetic diagnosis in the context of a known syndrome, contributing to a better comprehension of the complex transcriptional regulation of *TRPS1* and TRPS ethiopathogenesis.

## Background

Trichorhinophalangeal syndrome (TRPS) is a complex malformative disorder with autosomal dominant inheritance that is characterised by distinctive craniofacial and skeletal abnormalities [1]. TRPS patients generally present slow-growing and sparse scalp hair, medially thick and laterally thin eyebrows, a bulbous pear-shaped nose, a long flat philtrum, a thin upper vermilion border, large protruding ears [2], and bone abnormalities including mild to severe brachydactyly, cone-shaped epiphyses, hip dysplasia and short stature [2]. Malformations of inner organs have also been reported [3].

Three TRPS types can be distinguished at the clinical and molecular levels: TRPS I, II and III [1]. TRPS I (OMIM 190350) occurs as a consequence of inactivating mutations or chromosomal abnormalities that delete or disrupt the *TRPS1* gene [1,4-7]. TRPS II (OMIM 150230) is a contiguous gene syndrome caused by heterozygous deletions in 8q23.3–q24.11 involving the *TRPS1* and *EXT1* genes [8,9]. TRPS II is phenotypically distinguished from TRPS I by the presence of multiple cartilaginous exostoses and other less frequent features, such as intellectual disability, lax skin and a tendency toward bone fractures. Finally, TRPS III (OMIM 190351) is the result of missense mutations in the region of *TRPS1* that encodes a GATA-type zinc finger domain [2,6,10]. The primary clinical difference between TRPS I and TRPS III is in the severity of the skeletal abnormalities, especially brachydactyly and short stature [11].

The high dosage sensitivity of the *TRPS1* gene is underscored by mosaicism in some reported cases [9]. Moreover, a perturbation of *TRPS1* expression was previously hypothesised as causative in three TRPS II patients. These patients carried deletions at chromosome 8q24, which encompassed only the *EXT1* gene. In two of them, the proximal breakpoint was established and occurred at approximately 99.3 and 600–800 kb, respectively, from the *TRPS1* 5′ end [12-14]. Furthermore, a familial translocation t(8;13)(q23.3;q21.31), which did not disrupt *TRPS1* and co-segregated with TRPS I, was recently described [15]. In this case, the translocation resulted in the disruption of a transposon type I element, located at 87 kb from the *TRPS1* 5′ end, and in the simultaneous relocation of a non-coding conserved VISTA enhancer element from 13q21.31 within the *TRPS1* 5′ region, apparently leading to an increase in *TRPS1* gene expression in the translocation carriers [15].

In the current study, we report on a proband with a t(2;8)(p16.1;q23.3) *de novo* reciprocal chromosomal translocation who exhibits peculiar clinical features which mainly overlap with TRPS I. This is the second case in which a translocation breakpoint (bkp) does not interrupt the *TRPS1* gene and is not associated with its deletion. As in the previous report, identification of the bkps at nucleotide resolution suggests that, first the disruption and possibly the removal of *TRPS1 cis* regulatory elements and then the relocation of a conserved VISTA enhancer element nearby the *TRPS1* 5′ end, may be the cause of the proband’s unusual phenotype.

## Case presentation

### Clinical report

The proband is a 57-year-old Caucasian woman, born after an uncomplicated pregnancy to non-consanguineous healthy parents. The auxological parameters at birth were on the 50th percentile.

During childhood and adolescence growth was normal until a final height of 165 cm was reached, consistent with the proband’s midparental target height (167 cm). Psychomotor development was normal. Menarche occurred at 14 years, and menses have always been regular until menopause, which occurred at 45 years after surgery for hysterectomy. The proband showed no hair growth from early childhood and this rapidly progressed to alopecia universalis (i.e., absence of eyebrows and, after puberty, absence of pubic and axillary hair).

After the age of 15 years, the proband experienced several episodes of falls without loss of consciousness, but all neurological examinations performed (MRI, EMG and EEG) appeared normal. Echocardiogram revealed prolapse of both mitral valve leaflets and slight mitral regurgitation. After regular cardiologic follow-up, valve replacement was performed at the age of 45 years. Intraoperative findings revealed thickened mitral valve leaflets with the appearance of myxomatous degeneration, as confirmed by histological analysis. In the same year, the proband had a hysterectomy due to the presence of several fibroids, but the ovaries were preserved. Before surgery, she also suffered from a severe uterine prolapse.

We saw the proband for the first time at the age of 51, as she was referred to an endocrinology outpatient clinic because of hyperparathyroidism related to vitamin D deficiency. Endocrinology and genetic analyses were performed, with the aim of confirming a diagnosis of familial hyperparathyroidism based on the primary hyperparathyroidism of her mother. No germ-line mutations of the multiple endocrine neoplasia I (*MEN1*) gene were observed. Consequently, a diagnosis of tertiary hyperparathyroidism resulting from autonomous activity of the parathyroid glands, related to long-standing vitamin D deficiency, was raised. In addition, high levels of glycosylated haemoglobin (8%; reference range 4.5–6.5%) were observed, and the proband underwent metformin therapy. Bone mineral density evaluated by dual energy X-ray absorptiometry (Hologic) revealed vertebral and femoral osteoporosis (lumbar spine T score of −3.5 and femoral neck T score of −2.5), which was considered secondary to the endocrinological problems. There was no history of urolithiasis or nephrocalcinosis. Abdominal ultrasound analysis was normal, and only a nephroptosis of the right kidney was apparent. In addition, the proband’s mother referred a very high pain threshold in her daughter, who, for example, never required analgesia after surgery. The proband exhibited an important visual impairment characterised by severe astigmatism, cataracts and photophobia.

Physical examination revealed several craniofacial anomalies suggestive of a genetic disease: universal alopecia, deep-set eyes, bulbous pear-shaped nose, elongated philtrum, thin upper lip, high-arched palate, and large and prominent ears. The proband referred that her craniofacial dysmorphisms had been partially corrected by plastic surgery at the age of 43 years, particularly in the periorbital, maxillary, and mandibular areas, suggesting the presence of severe facial bone anomalies. However, we cannot confirm these information as she would not grant permission for us to see pre-surgery facial photographs and the surgery report was not available. Finally, she showed bilateral valgi and flat feet, and an anxious and obsessive psychological attitude. Cognitive function was not formally evaluated.

The observed clinical findings suggested a diagnosis of Trichorhinophalangeal syndrome. Therefore, a radiological study of the skeleton was performed; this revealed hypoplastic mandibular condyles and severe scoliosis. No abnormalities were observed in hands (Additional file 1: Figure S1), arms, legs and pelvis. At age 56 an infiltrating ductal carcinoma, sized 11 cm, was identified in the right breast, and was subsequently removed by mastectomy. Histological examination characterised the cancer as oestrogen-negative, and the proband is currently undergoing chemotherapy.

The proband does not accept the genetic basis for her condition, and denied permission to release her photos for publication.

### Methods

#### Cytogenetic analysis

Conventional cytogenetic analysis was performed on the proband and her healthy parents on QFQ-banded metaphases prepared from peripheral blood lymphocytes using standard procedures. The karyotypes were described in accordance with ISCN (2009) [16].

#### High-resolution array comparative genomic hybridisation (CGH) analysis

Genomic DNA was extracted from whole blood using the GenElute Blood Genomic DNA kit (Sigma-Aldrich, St. Louis, MO). Array CGH analysis was performed using the Human Genome CGH Microarray Kit 244 K (Agilent Technologies, Palo Alto, CA). From both test and normal reference samples, 3 μg of DNA were processed according to the manufacturer’s instructions. Images were captured using the Agilent Feature Extraction 9.1 software and chromosomal profile was acquired using the ADM-2 algorithm provided by DNA Analytics software (v4.0) (Agilent Technologies).

#### Fluorescence in situ hybridisation (FISH) analysis

FISH mapping of the bkps was performed using BAC clones, targeting the chromosomal bkp regions 2p16.1-p15 and 8q23.3-q24.1, as probes. The clones were provided by Invitrogen ltd (Carlsbad, CA, USA), and selected by consulting the UCSC Genome Browser Database (University of California Santa Cruz, reference genome assembly GRCh37/hg19) [17]. All BAC clone DNAs were labelled by nick-translation with Cy3-dUTP (GE Healthcare, Little Chalfont, Buckinghamshire, UK) and the FISH protocol described by Lichter and Cremer [18] was followed, with minor modifications.

The 2p breakpoint was further narrowed down by means of three contiguous overlapping 15-kb long-range polymerase chain reaction (LR-PCR) products as probes (LRP I, II, III). The fragments were amplified by LR-PCR using the TaKaRa LA Taq™ kit (Takara Bio Inc., Shiga, Japan) using approximately 100 ng of BAC clone CTD-2562H20 as template, and then labelled by random priming (Prime-It Fluor Fluorescence labelling kit, Stratagene, Amsterdam, Netherlands). The primer pairs are shown in Additional file 2: Table S1.

#### Amplification of the junction fragments

To localize the breakpoints at nucleotide level, sequence-specific LR-PCR was carried out. Oligonucleotides and amplification conditions used to amplify the derivative chromosome der(2) and der(8) junction fragments are shown in Table 1 and Additional file 3: Table S2. LR-PCRs were performed using the TaKaRa LA Taq™ kit (Takara Bio Inc.), and the resulting junction fragments were sequenced using the Big Dye® Terminator v.3.1 Cycle Sequencing kit (Applied Biosystems, Foster City, CA). Sequences were then aligned to the human reference genome sequence (human genome assembly GRCh37/hg19), analysed with the ChromasPro 1.5 software (Technelysium Pty Ltd., Tewantin QLD, Australia), and submitted to GenBank (http://www.ncbi.nlm.nih.gov/WebSub). *In-silico* analysis of bkp regions was performed by consulting the UCSC Genome Browser and the VISTA Enhancer Browser Database [19].

**Table 1:** Primers used for amplification of der(2) and der(8) junction fragments

***Fragment***	***Designation***	***Primer sequence (5′→3′)***	***Primer localization***^***a***^	***Annealing T(°C)***	***PCR size (bp)***
*Der(8) junction fragment*	AF130342-3FW	CCTTCTAGAGCAAATTCTTTTAGACCTTGA	chr8:116,981,340-116,981,369	62.4	632
	AC007131-5FW	CTCATGGTGTAGAATAGAAGCAGCAAGT	chr2:59,567,411-59,567,438		
*Der(2) junction fragment*	AF130342-1RW	GTTGACATCAGGACTTCAGGTAAATGAA	chr8:116,981,900-116,981,927	61.4	701
	AC007131-4RW	AATTTCTCCTTTATTCCTCTCCCCTTTC	chr2:59,568,122-59,568,149		

^a^Primer physical localization is based on GRCh37/hg19 human genome assembly.

#### Mutation screening

The entire coding sequence, intron-exon junctions and untranslated exons of the *TRPS1* gene (RefSeq Accession: NM_014112.4) were amplified for mutation screening by PCR using the AmpliTaq Gold® kit (Applied Biosystems). The primer pairs and amplification conditions are summarized in Additional file 4: Table S3. Sequencing was performed as described before.

### Results

Standard cytogenetic analysis revealed in the proband a *de novo* apparently balanced reciprocal chromosome translocation between the short arm of chromosome 2 and the long arm of chromosome 8 [t(2;8)(p15;q24.1)]. The proband’s parents had normal karyotypes. The subsequent high-resolution array CGH analysis excluded the presence in the proband of rare CNVs spanning the translocation bkp chromosomal bands or localized elsewhere in the genome.

**Figure 1:**
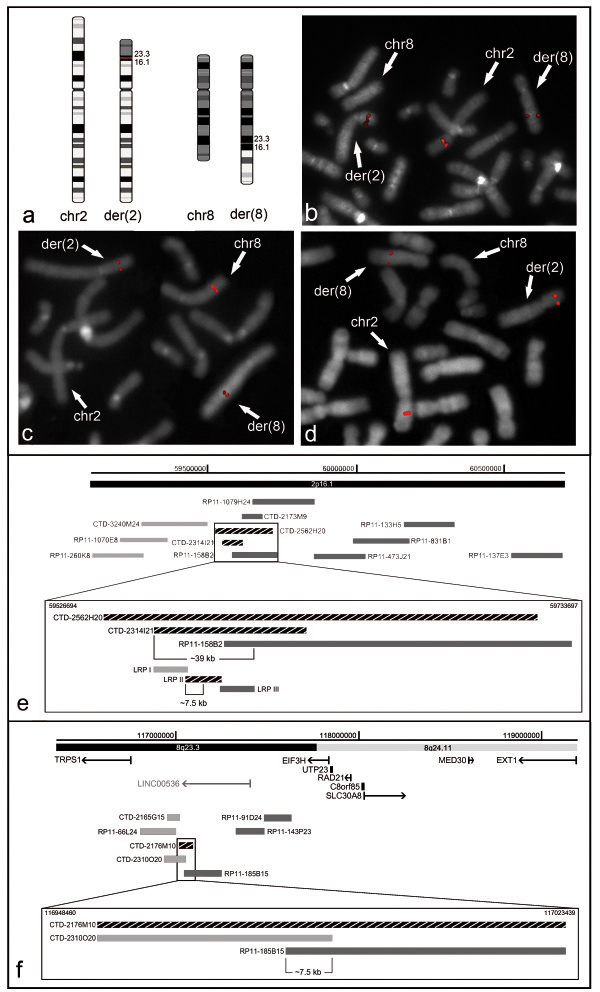
**Mapping of the breakpoint by FISH analysis. (a)** Ideograms illustrating the derivative chromosomes involved in the t(2;8)(p16.1;q23.3)dn, and the normal homologous chromosomes. **(b)** FISH with BAC clone CTD-2314I21, which spans the translocation bkp at 2p16.1, yields hybridisation signals of equal intensity on der(2) and der(8) chromosomes. **(c)** FISH with BAC clone CTD-2176M10, which spans the translocation bkp at 8q23.3, produces signals of comparable intensity on both derivative chromosomes. **(d)** FISH with long-range probe (LRP) II, which spans the translocation bkp at 2p16.1, shows a more intense signal on der(2) than on der(8). **(e and f)** Physical map of the genomic regions containing **(e)** the 2p16.1 bkp and **(f)** the 8q23.3 bkp, which includes the BAC clones and LRPs used for the FISH analysis. The probes mapping at chromosome 2 **(e)** and chromosome 8 **(f)**, which show a hybridization signal on der(2), are indicated in grey, whereas those hybridizing on der(8) in light grey. The probes spanning the bkp regions are indicated by striped black rectangular shapes, the known UCSC genes are shown in black, and the *LINC00536* in light grey (human genome assembly GRCh37/hg19).

As we hypothesized that the rearrangement was the main cause of the proband’s phenotype, we refined the bkps by FISH mapping. The chromosome 2 bkp was mapped at 2p16.1 (Figure 1a), within the region spanned by probe CTD-2562H20, and refined by CTD-2314I21 (GenBank accession number AC010479.5) (Figure 1b), whereas the chromosome 8 bkp was identified by the clone CTD-2176M10 (chr8:116,948,460-117,023,439) at 8q23.3 (Figure 1a,c). Therefore, based on BAC FISH data, the der(2) and der(8) bkps were mapped within a region of about 39 kb (chr2:59548766–59587488) and 7.5 kb (chr8:116978981–116986481), respectively (Figure 1e,f). The location of the 2p16.1 bkp was further narrowed down using three contiguous overlapping 15-kb LR-PCR products as FISH probes (LRP I, II, III) (Figure 1e). The LRP II produced weak but clear signals on both derivative chromosomes that were more intense on der(2), suggesting that the bkp was localized within the telomeric 7.5-kb fragment of LRP II target region (chr2:59563376–59570875) (Figure 1d,e).

**Figure 2:**
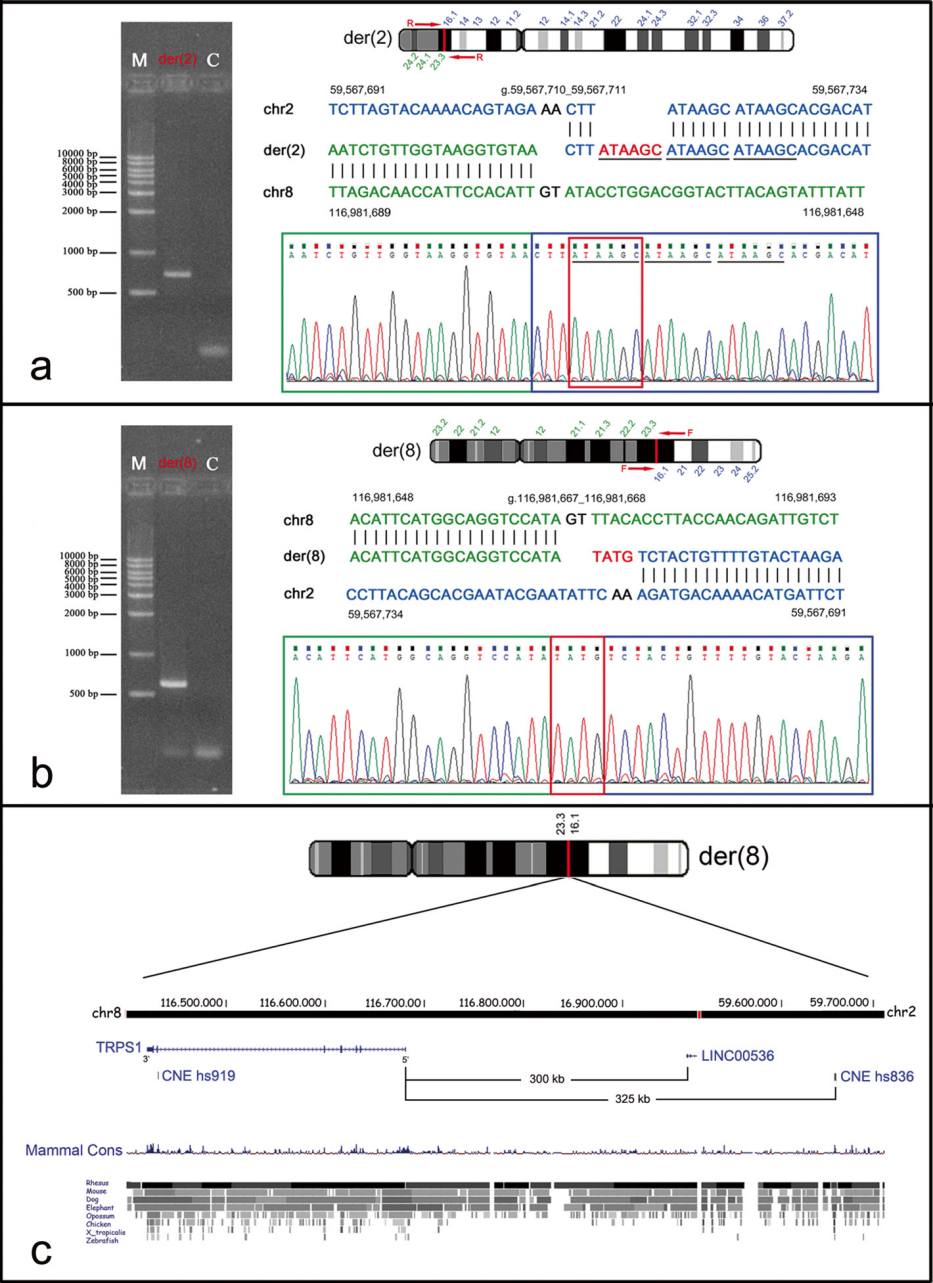
**Gel electrophoresis of der(2) and der(8) junction fragments, and bkp sequence alignments.** (left side) The der(2) **(a)** and the der(8) **(b)** bkp junction fragments were amplified with the primer pairs AF130342-1RW/AC007131-4RW, and AF130342-3FW/AC007131-5FW, respectively, producing a ~700 and ~600 bp fragments. (right side) Electropherograms of der(2) **(a)** and der(8) **(b)** bkp junction fragments are shown with the respective alignments against the reference sequence. Positions of the bkps at DNA sequence level are indicated (human genome assembly GRCh37/hg19). Chromosome 8 sequence is in green; sequence related to chromosome 2 in blue; bases lost upon rearrangement in black; bases inserted *de novo* in red. The triple-tandem repeats of the ATAAGC *de novo* acquired hexamer are underlined with a solid line. The GenBank accession numbers of the submitted der(2) and der(8) junction fragment sequences are KJ561173 and KJ561174, respectively. **(c)** Ideogram of chromosome der(8), showing the relocation of the conserved non-coding element (CNE) VISTA enhancer hs836 at an approximate distance of 325 kb from the *TRPS1* 5′ region as a result of the translocation. The image is a modification of a version obtained from the UCSC Genome Browser [17].

The breakpoint junction fragments were then amplified and sequenced. Sequence alignments showed on der(2) the loss of two AA bases at position g.59,567,711_59,567,712, and the duplication of the ATAAGC hexamer at position g.59,567,716_59,567,721 (Figure 2a). Similarly, on der(8) a 2-bp GT deletion at position g.116,981,668_116,981,669, where the missing T is highly conserved [20], was detected as well as a *de novo* 4 bp TATG insertion at position g.116,981,668_116,981,671 (Figure 2b), indicating that the rearrangement is not completely balanced. The 8q23.3 breakpoint was precisely located at position g.116,981,667_116,981,668 within the IVS10 of the long intergenic non-coding RNA (lincRNA) 536 (*LINC00536*, chr8:116,962,736-117,337,297), at approximately 300 kb from the *TRPS1* 5′ end (Figure 2c and Additional file 5: Figure S2B). The 2p16.1 breakpoint was localized at position g.59,567,710_59,567,711 within a 418-bp LINE sequence type 2 (L2a, chr2:59,567,631-59,568,048) (Additional file 5: Figure S2A). This bkp is flanked distally, at 1.1 Mb, by the *FANCL* (Fanconi anemia, complementation group L) gene, and proximally, at 1.1 Mb, by the *BCL11A* (B-cell CLL/lymphoma 11A) gene. In addition, about 25 kb distally to the der(2) bkp, we noticed the presence of the conserved non-coding element (CNE) (VISTA enhancer element hs836 at position chr2:59,540,641-59,541,193). Notably, this VISTA CNE showed a specific expression pattern in transgenic mouse embryos, demonstrating its activity in facial mesenchyme development (http://enhancer.lbl.gov/cgi-bin/imagedb3.pl?form=presentation&show=1&experiment_id=element_836&organism_id=1). As a result of the translocation, the enhancer sequence has been relocated to a new position, at approximately 325 kb from the *TRPS1* 5′ end (Figure 2c).

Additional mutations within *TRPS1* were excluded in the proband by sequence analysis.

## Discussion

We have herein described a proband with an unusual presentation of TRPS I, who was found to carry a *de novo* reciprocal translocation involving the 2p16.1 and 8q23.3 chromosomal bands. The proband represents an atypical case as she does not bear a microdeletion involving *TRPS1* or a mutation in the gene coding sequence, and characterisation of her translocation bkps excluded the disruption of the *TRPS1* gene. The sequence analysis of the bkp junction fragments, however, precisely located the 8q23.3 bkp on chromosome der(8) at approximately 300 kb from the *TRPS1* 5′ end, thus pointing to *TRPS1* as the gene responsible for the proband’s TRPS I phenotype. In addition, nucleotide base pair additions and deletions were detected, thus indicating that the translocation was likely mediated by the Non-Homologous End Joining (NHEJ) mechanism [21].

As previously reported in a t(8;13)(q23.3;q21.31) familial translocation co-segregating with TRPS I [15], in the present subject neither bkp occurs within a coding region. However, the rearrangement interrupts and changes the positions of gene regulatory elements with respect to their original gene targets. We suggest that the disruption and the possible removal of *TRPS1 cis* regulatory elements, such as the *LINC00536*, might be causative, consistent with the previous hypotheses in a few reported patients [12-15]. To date, neither the precise biological function nor the target gene/s of *LINC00536* are known. However, as lincRNAs are key regulators of diverse cellular processes [22], we hypothesize that *LINC00536* disruption might have contributed to the onset of the proband’s clinical phenotype. Interestingly, the 8q23.3 bkp likely interrupts a putative enhancer region [23,24] (Additional file 5: Figure S2B), and maps within a genomic region where DNA sequences interacting with transcription factors, identified by ChIP-seq experiments, have been localized [25,26] (Additional file 5: Figure S2B).

Notably, the 2p16.1 bkp was positioned at approximately 25 kb proximal to the conserved VISTA enhancer element hs836, whose activity in facial mesenchyme development has been substantiated by gene reporter assays in mouse embryos [27]. Similarly to what recently reported by David et al. [15], in the present subject the enhancer element was relocated by the translocation in the vicinity of the *TRPS1* 5′ end, thus suggesting a possible “enhancer adoption”, a mechanism recently described by Lettice et al. [28], which might have perturbed *TRPS1* expression in the facial region during embryonic development. In agreement with this hypothesis, David et al. [15] detected an apparently *TRPS1* overexpression in the translocation carriers compared with controls.

There are many potential consequences resulting from chromosomal rearrangements that could lead to position effects and thus cause human disease. These include the moving away of an enhancer or a locus control region from its gene, the juxtaposition of a gene with a regulatory element from another gene, and the removal of a boundary element or a long-range insulator [28,29]. On this basis, the unique reshaping of regulatory elements occurring in the proband could have deregulated the expression of *TRPS1*, thus leading to the observed clinical phenotype. However, we could not demonstrate any alteration in *TRPS1* expression as preliminary *TRPS1* gene expression assays, performed from the proband’s peripheral blood, gave inconclusive results due to very different *TRPS1* expression levels found in controls (Additional file 6: Figure S3).

The peculiar chromosome rearrangement herein described could also explain the differences of both the craniofacial and skeletal abnormalities of our proband from those normally found in TRPS patients. Indeed, important skeletal features of TRPS I such as short stature, brachydactyly, and the pathognomonic abnormality of cone-shaped epiphyses were not observed (Additional file 1: Figure S1). In addition, the proband exhibited the main TRPS facial dysmorphism as well as bone anomalies from early infancy; but these were so severe as to require plastic surgery. Such severe abnormalities, possibly accounted for by significant *TRPS1* deregulation in the facial mesenchyme during development, are not frequently associated with TRPS. The proband also displayed an important scoliosis and alopecia universalis, clinical findings that are markedly more severe than those normally observed in TRPS patients.

Genotype-phenotype correlations were hard to assess for a few clinical signs, namely minor mental impairment, diabetes and a mild clinical presentation of connective tissue disorder (mild joint laxity, severe astigmatism, renal ptosis, and uterine prolapses). These symptoms may be caused by mutations of unknown genes, although a contribution of the chromosomal rearrangement in deregulating breakpoint-neighbouring genes cannot be ruled out. Similarly, we cannot exclude the possible influence of the translocation, via altered gene expression, on the development of breast cancer. Indeed, *TRPS1* gene overexpression in more than 90% of breast cancers has been reported [30].

## Conclusions

To conclude, this case report, presenting a new case of association of TRPS I-like phenotype with a reciprocal chromosomal translocation which does not disrupt the *TRPS1* coding sequence, increases the number of TRPS patients whose pathologic phenotype is caused by a functional disturbance of *TRPS1*. The clinical and genetic characterisation of the present subject allowed us to make a genetic diagnosis in the context of a known syndrome, contributing to expand the TRPS phenotypic spectrum. In addition, this study provides a further comprehension of the complex transcriptional regulation of developmental genes such as *TRPS1.* The identification and mapping at nucleotide level of novel genomic alterations in TRPS patients will be necessary to better understand the pathogenesis of Trichorhinophalangeal syndrome and the regulation of *TRPS1*.

## Consent

Written informed consent was obtained from the proband for publication of this Case Report and any accompanying images. A copy of the written consent is available for review by the Editor of this journal.

## Abbreviations

Array CGH: Array comparative genomic hybridisation; BAC: Bacterial artificial chromosome; ChIP: Chromatin immunoprecipitation; EEG: Electroencephalography; EMG: Electromyography; FISH: Fluorescence in Situ hybridisation; bkp: Breakpoint; LRP: Long-range PCR probe; MRI: Magnetic resonance imaging; NHEJ: Non-homologous end joining; PCR: Polymerase chain reaction; RT-qPCR: Reverse transcription quantitative PCR; TRPS: Trichorhinophalangeal syndrome; UCSC: University of California Santa Cruz.

## Competing interest

None of the authors have any conflict of interests with the content of this manuscript.

## Authors’ contributions

FP had a main role in conception and design of the study, and in the analysis and interpretation of the data. CM contributed to the study design and interpretation of data. CC and CM performed conventional cytogenetic and FISH experiments, and interpreted the data. CM and TS performed expression analyses, and interpreted the data. CM performed array CGH analysis. CM, PC, and BI performed the *TRPS1* mutation screening. BI performed the bkp analysis, and interpreted the data. PAI, PM, and GG performed clinical investigations at different times and reviewed all clinical records. CM and FP drafted the manuscript. FP, CC, and LL took part in critical revision of the manuscript. All authors approved the final version of the report.

## Pre-publication history

The pre-publication history for this paper can be accessed here:

http://www.biomedcentral.com/1471-2350/15/52/prepub

## Supplementary Material

Additional file 1: Figure S1 Radiograph of proband’s hands. Radiograph of left and right hand, which lacks the pathognomonic TRPS abnormality of cone-shaped epiphyses. Click here for file

Additional file 2: Table S1 List of primers used to amplify Long-range PCR fragments on chromosome 2. Click here for file

Additional file 3: Table S2 List of primers used for amplification of chromosome der(2) and der(8) junction fragments. Click here for file

Additional file 4: Table S3 List of primers and amplification conditions used to perform *TRPS1* mutational screening. Click here for file

Additional file 5: Figure S2 *In silico* analysis of the genomic regions containing the translocation bkps. *In silico* analysis of the genomic regions containing the 2p16.1 and 8q23.3 bkps. **(a)** The 2p16.1 bkp interrupts the repeat element L2a (red arrow) corresponding to a LINE sequence, and is localised approximately 25 kb from the conserved non-coding element (CNE) VISTA enhancer hs836. **(b)** The 8q23.3 bkp interrupts the lincRNA *LINC00536* as well as a putative enhancer region (in orange), and is located in a region with numerous predicted regulatory sequences identified by ChIP-Seq experiments (in dark grey). The coloured bars represent the putative regulatory sequences identified by a probabilistic Hidden Markov Model (HMM) applied to HMEC cells (Human Embryonic Stem Cell). The image is a modification of a version obtained from the UCSC Genome Browser (human genome assembly GRCh37/hg19) [17]. Click here for file

Additional file 6: Figure S3 Reverse transcription quantitative PCR (RT-qPCR) expression analysis of *TRPS1.* RT-qPCR expression analysis of *TRPS1*. **(a)** Relative expression level of the *TRPS1* transcript in blood lymphocytes of the proband compared to 10 controls from normal individuals, by using TaqMan gene expression assays. The amounts of *TRPS1* mRNA (TaqMan assay ID Hs00936363_m1) were calculated using the 2-∆∆Ct method and expression values were normalised to the internal control gene *GAPDH* (TaqMan assay ID Hs99999905_m1) **(b)** Similar results were obtained by using the *TBP* housekeeping gene (TaqMan assay ID Hs99999910_m1) The expected ΔΔCt ratio is ≅1 when both alleles are expressed, and 0.5 when only one allele is expressed. x-axis: a dark grey bar indicates the proband (PB), whereas light grey bars indicate controls (C1–C10). y-axis: average of three recorded expression levels for each sample; the proband’s value was set to 1. Statistical analysis was performed by two-tailed Student’s *t* test and significance was considered at *P* < 0.01. Click here for file
