# Supplementary figures and images for: New case of trichorinophalangeal syndrome-like phenotype with a de novo t(2;8)(p16.1;q23.3) translocation which does not disrupt the TRPS1 gene
Source: BMC Med Genet. 2014 May 2;15:52. doi: 10.1186/1471-2350-15-52 (PMC4081657; doi:10.1186/1471-2350-15-52)

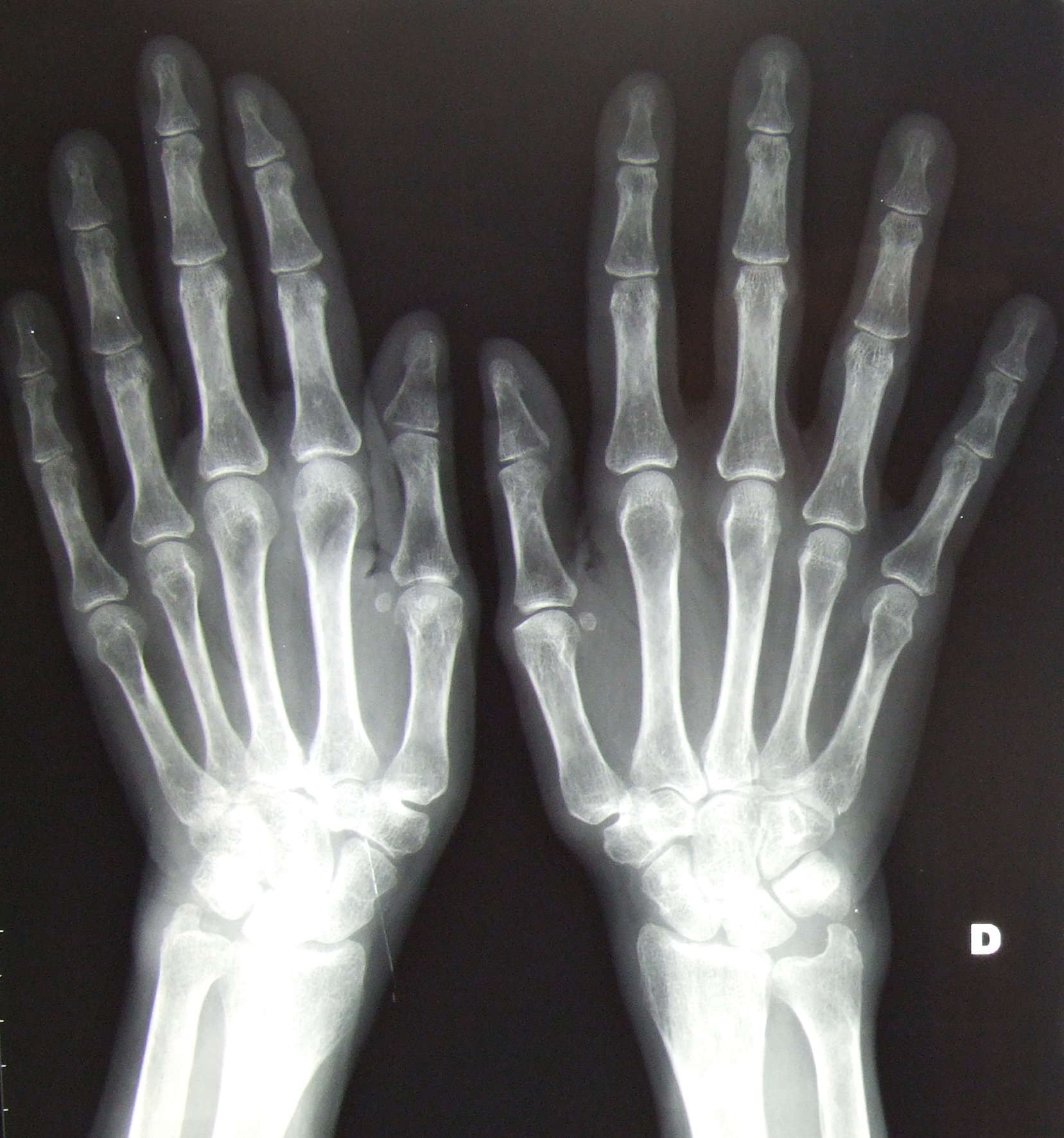

Supplement: Additional file 1: Figure S1 — Radiograph of proband’s hands. Radiograph of left and right hand, which lacks the pathognomonic TRPS abnormality of cone-shaped epiphyses. [file 1471-2350-15-52-S1.tiff]

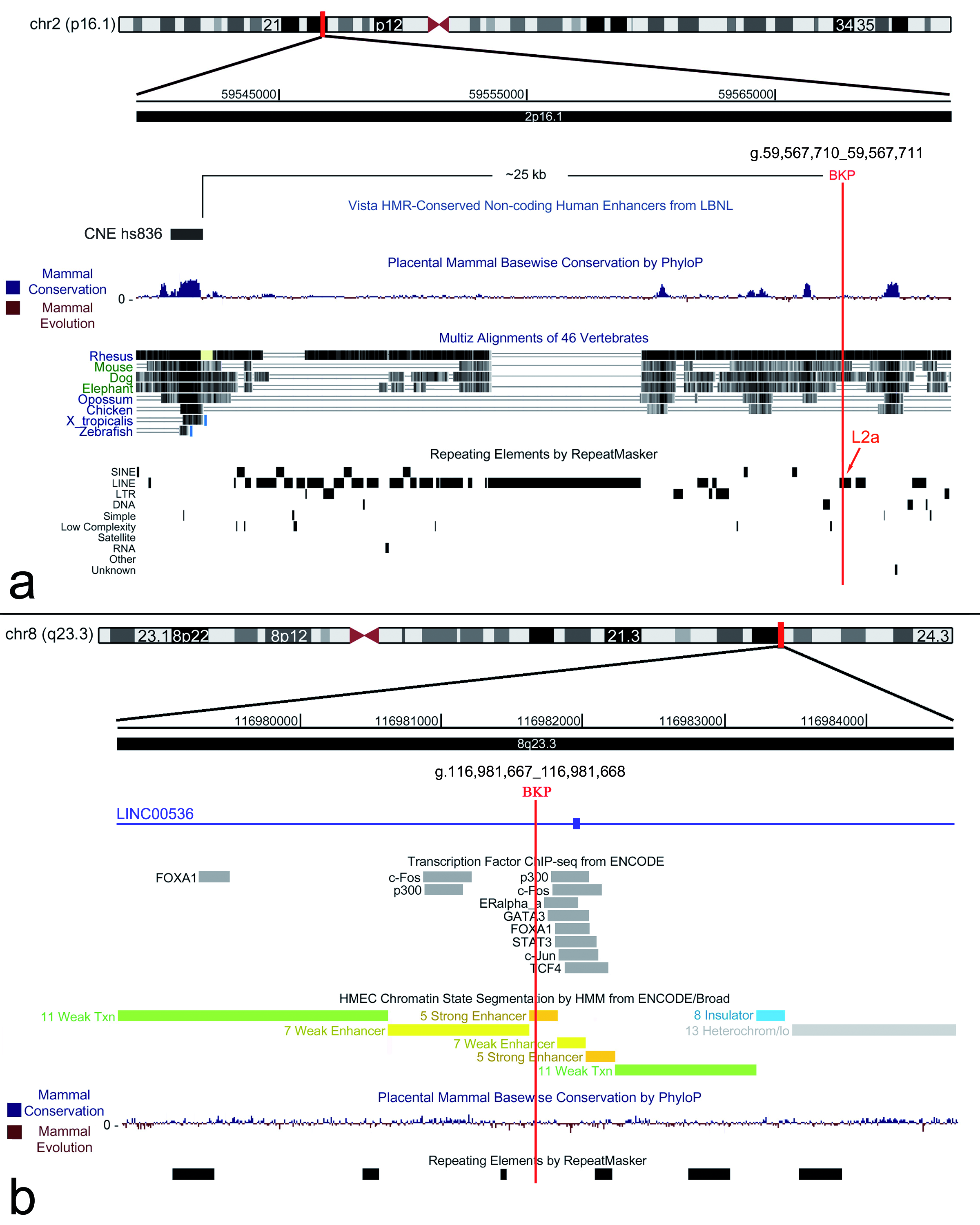

Supplement: Additional file 5: Figure S2 — In silico analysis of the genomic regions containing the translocation bkps. In silico analysis of the genomic regions containing the 2p16.1 and 8q23.3 bkps. (a) The 2p16.1 bkp interrupts the repeat element L2a (red arrow) corresponding to a LINE sequence, and is localised approximately 25 kb from the conserved non-coding element (CNE) VISTA enhancer hs836. (b) The 8q23.3 bkp interrupts the lincRNA LINC00536 as well as a putative enhancer region (in orange), and is located in a region with numerous predicted regulatory sequences identified by ChIP-Seq experiments (in dark grey). The coloured bars represent the putative regulatory sequences identified by a probabilistic Hidden Markov Model (HMM) applied to HMEC cells (Human Embryonic Stem Cell). The image is a modification of a version obtained from the UCSC Genome Browser (human genome assembly GRCh37/hg19) [17]. [file 1471-2350-15-52-S5.tiff]

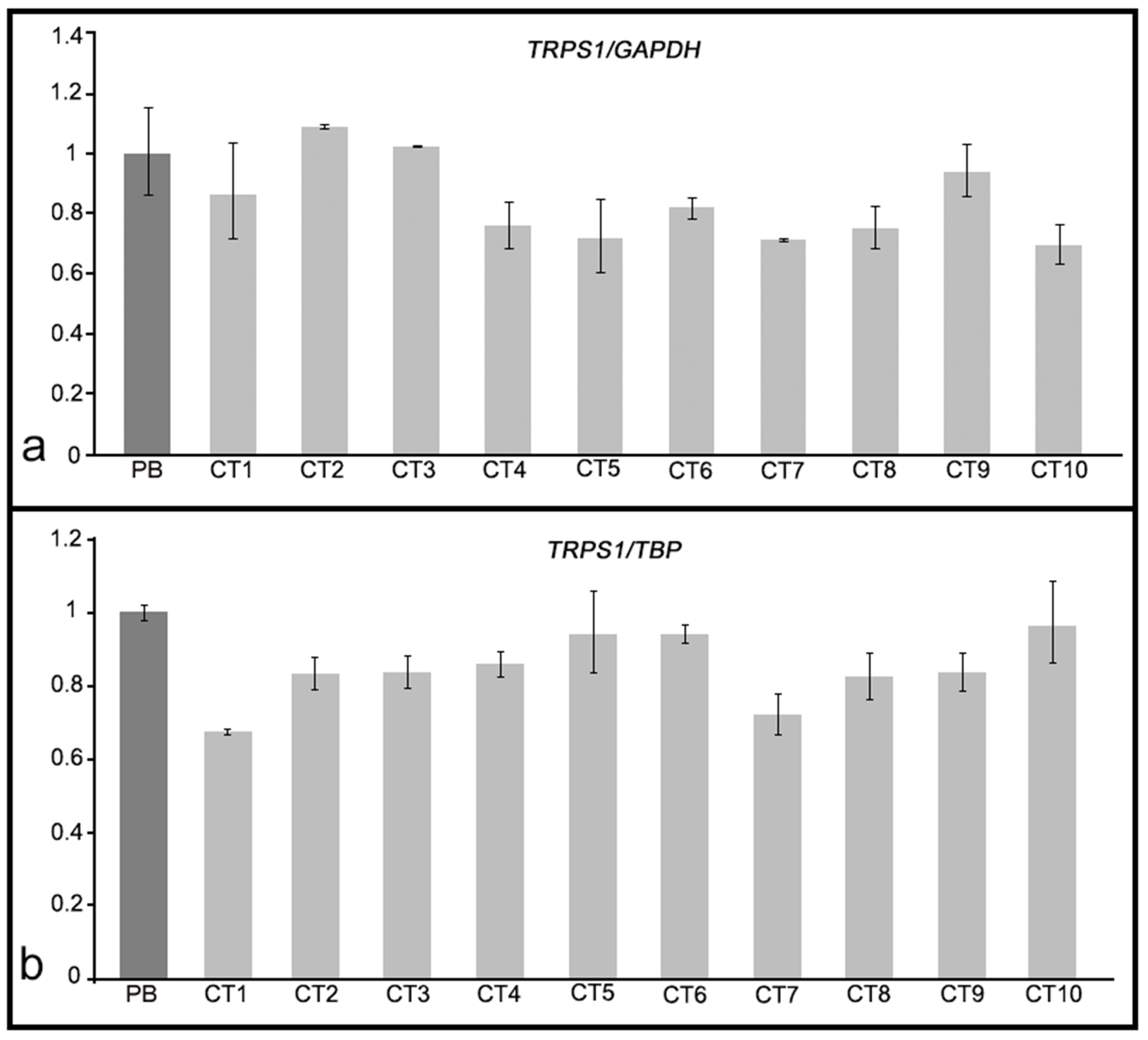

Supplement: Additional file 6: Figure S3 — Reverse transcription quantitative PCR (RT-qPCR) expression analysis of TRPS1. RT-qPCR expression analysis of TRPS1. (a) Relative expression level of the TRPS1 transcript in blood lymphocytes of the proband compared to 10 controls from normal individuals, by using TaqMan gene expression assays. The amounts of TRPS1 mRNA (TaqMan assay ID Hs00936363_m1) were calculated using the 2-∆∆Ct method and expression values were normalised to the internal control gene GAPDH (TaqMan assay ID Hs99999905_m1) (b) Similar results were obtained by using the TBP housekeeping gene (TaqMan assay ID Hs99999910_m1) The expected ΔΔCt ratio is ≅1 when both alleles are expressed, and 0.5 when only one allele is expressed. x-axis: a dark grey bar indicates the proband (PB), whereas light grey bars indicate controls (C1–C10). y-axis: average of three recorded expression levels for each sample; the proband’s value was set to 1. Statistical analysis was performed by two-tailed Student’s t test and significance was considered at P < 0.01. [file 1471-2350-15-52-S6.tiff]
