# Supplementary material for: New case of trichorinophalangeal syndrome-like phenotype with a de novo t(2;8)(p16.1;q23.3) translocation which does not disrupt the TRPS1 gene
Source: BMC Med Genet. 2014 May 2;15:52. doi: 10.1186/1471-2350-15-52 (PMC4081657; doi:10.1186/1471-2350-15-52)
Supplement: Additional file 2: Table S1 — List of primers used to amplify Long-range PCR fragments on chromosome 2. [file 1471-2350-15-52-S2.doc]

**Table S1 Primers used to amplify Long-range PCR (LRP) fragments on chromosome 2**

| ***Fragment*** | ***Designation*** | ***Primer sequence (5’🡪3’)*** | ***Primer localization^a^*** | ***Annealing T(°C)*** | ***PCR size (bp)*** |
| --- | --- | --- | --- | --- | --- |
| *LRP I* | AC007131-1Fw  AC007131-1Rev | TCCAATCAAGCATACACAAAACACTTCT  ACAATCATTTTACTTGCTTCCTCTCAGC | chr2:59,549,048-59,549,075  chr2:59,563,657-59,563,684 | 60.7 | 14,637 |
| *LRP II* | AC007131-2Fw  AC007131-2Rev | TCAGAAACCTATGGAATAGAGGATTGGA  TTTCTCAATGAAGTGTGCATTAGTTGGT | chr2:59,563,376-59,563,403  chr2:59,578,835-59,578,862 | 60.7 | 15,487 |
| *LRP III* | AC007131-3Fw  AC007131-3Rev | ATTTGTCAGGACCACATTTTTCCATTAC  ATACCACAGTTGCCTTCCTAAGAAACCT | chr2:59,578,780-59,578,807  chr2:59,593,831-59,593,858 | 61.4 | 15,079 |
| ^a^Primer physical localization is based on GRCh37/hg19 human genome assembly. | | | | | |
