# Supplementary material for: New case of trichorinophalangeal syndrome-like phenotype with a de novo t(2;8)(p16.1;q23.3) translocation which does not disrupt the TRPS1 gene
Source: BMC Med Genet. 2014 May 2;15:52. doi: 10.1186/1471-2350-15-52 (PMC4081657; doi:10.1186/1471-2350-15-52)
Supplement: Additional file 3: Table S2 — List of primers used for amplification of chromosome der(2) and der(8) junction fragments. [file 1471-2350-15-52-S3.doc]

**Table S2** **Additional primers used for amplification of chromosome der(2) and der(8) junction fragments**

| ***Designation*** | ***Primer sequence (5’🡪3’)*** | ***Primer localization^a^*** | ***Annealing T(°C)*** | ***PCR size (bp)*** |
| --- | --- | --- | --- | --- |
| AF130342-1FW  AC007131-4FW | CTTGATGACTGGATGAAGAGACAATAGG  GCTTGTTAACTTTTACTCTGGGCACAAA | chr8:116,979,741-116,979,768  chr2:59,566,980-59,567,007 | 62.9 | 2,658 |
| AF130342-2FW  AC007131-4FW | TGAAAATGGTCCAGCCTTTATTTATGAC  GCTTGTTAACTTTTACTCTGGGCACAAA | chr8:116,981,622-116,981,649  chr2:59,566,980-59,567,007 | 61.4 | 777 |
| ^a^Primer physical localization is based on GRCh37/hg19 human genome assembly. | | | | |
