# Supplementary material for: New case of trichorinophalangeal syndrome-like phenotype with a de novo t(2;8)(p16.1;q23.3) translocation which does not disrupt the TRPS1 gene
Source: BMC Med Genet. 2014 May 2;15:52. doi: 10.1186/1471-2350-15-52 (PMC4081657; doi:10.1186/1471-2350-15-52)
Supplement: Additional file 4: Table S3 — List of primers and amplification conditions used to perform TRPS1 mutational screening. [file 1471-2350-15-52-S4.doc]

**Table S3 Oligonucleotides and amplification conditions used to perform *TRPS1* mutational screening**

| ***Region*** | ***Designation*** | ***Primer sequence (5’🡪3’)*** | ***Annealing T(°C)*** | ***PCR size (bp)*** |
| --- | --- | --- | --- | --- |
| *Exon 1* | TRPS1-Ex1F  TRPS1-Ex1R | TGAGGTCCTGACAAGCGATA  GCCATGAACCGTCTTCTGTT | 57.2 | 355 |
| *Exon 2* | TRPS1-Ex2F | TCTTTCAGGCAGCCTATGAT | 55.2 | 322 |
|  | TRPS1-Ex2R | TGTGCCAAGAACTTTTCCAG |  |  |
| *Exon 3* | TRPS1-Ex3.1F | TTCCAGATTCAACTGTATCTCTGT | 58 | 292 |
|  | TRPS1-Ex3.1R | CCTTCTTACTGCTAGAAGATGGAT |  |  |
|  | TRPS1-Ex3.2F | TACGGATCAGAGTGATGCTG | 56.2 | 392 |
|  | TRPS1-Ex3.2R | CTTGACAATTGGCTTGACCA |  |  |
|  | TRPS1-Ex3.3F | GGGGACTCACTGGAGACAAA | 58.3 | 391 |
|  | TRPS1-Ex3.3R | GGCTGAACTGCACCATGTTA |  |  |
|  | TRPS1-Ex3.4F | GACTGCATAACCGCACCAG | 60.1 | 282 |
|  | TRPS1-Ex3.4R | CCTGTCTGGTACTGGGACCT |  |  |
| *Exon 4* | TRPS1-Ex4.1F | TGGAATACTTGGCAGTACCC | 57.5 | 391 |
|  | TRPS1-Ex4.1R | TGTCATCTCCTGCTTTGACTG |  |  |
|  | TRPS1-Ex4.2F | GTCCATCCCTGCACTTCAAT | 57.2 | 382 |
|  | TRPS1-Ex4.2R | CTTCTTTTTAGCCCCACTCG |  |  |
|  | TRPS1-Ex4.3F | ATAAGCTTTCCAGGGGCTCT | 58.3 | 384 |
|  | TRPS1-Ex4.3R | GCAGAAGCAAGAGTGCACAG |  |  |
|  | TRPS1-Ex4.4F | TCCGTTTGCTTGTAGAAAAAGT | 55.8 | 399 |
|  | TRPS1-Ex4.4R | CTCAACAATTCCCGGTTCAG |  |  |
| *Exon 5* | TRPS1-Ex5.1F | ACAACCCTTCAGAACGCTGTC | 59.5 | 350 |
|  | TRPS1-Ex5.1R | CTGGAACTCTCGGTCCAAAC |  |  |
|  | TRPS1-Ex5.2F | ATGGGAGAGCCAGTTTCTGA | 57.8 | 332 |
|  | TRPS1-Ex5.2R | AGGGCCCCAGACTTCTCT |  |  |
|  | TRPS1-Ex5.3F | GGGATAGTCCCAATGTGGAG | 58.7 | 273 |
|  | TRPS1-Ex5.3R | CTTCACACACAACACAATCCTG |  |  |
| *Exon 6* | TRPS1-Ex6.1F | AAGACAAAACTCCTGGGTTGAT | 57.6 | 300 |
|  | TRPS1-Ex6.1R | CACTGCAAGCCAGGGAATG |  |  |
| *Exon 7* | TRPS1-Ex7.1F | GCATGGTTTATATTTGTGAGGAA | 56.1 | 385 |
|  | TRPS1-Ex7.1R | ATCCAGAGTTTGGCTGACCA |  |  |
|  | TRPS1-Ex7.2F | TCCACTCCCCAGCCTAAGTA | 59.3 | 382 |
|  | TRPS1-Ex7.2R | CCCAGGAACGGAGAGCTTAT |  |  |
|  | TRPS1-Ex7.3F | CCACTTTTTGGACTTCCCTTT | 55.5 | 389 |
|  | TRPS1-Ex7.3R | ATGCCACAGTGCACACATTT |  |  |
|  | TRPS1-Ex7.4F | AAATGAAGGTCCCTTGAATGTA | 55.8 | 390 |
|  | TRPS1-Ex7.4R | TGTTGGATAAGGCAGGCTCT |  |  |
|  | TRPS1-Ex7.5F | GCACGGACAAATATGACTTCA | 54.8 | 300 |
|  | TRPS1-Ex7.5R | TCCATTCTTTCTCATTGACCA |  |  |
